# Mammography Use in Portugal: National Health Survey 2014

**DOI:** 10.5888/pcd14.170054

**Published:** 2017-10-19

**Authors:** Sofia Chkotua, Bárbara Peleteiro

**Affiliations:** 1 Institute of Public Health (ISPUP)–Epidemiology Research Unit (EPI Unit), Universidade do Porto, Porto, Portugal; 2 Departamento de Epidemiologia Clínica, Medicina Preditiva e Saúde Pública, Faculdade de Medicina, Universidade do Porto, Porto, Portugal

## Abstract

**Introduction:**

Understanding the patterns of mammography use and monitoring changes in use are essential to improving national health policy for breast cancer control. We aimed to describe the use of mammography in Portugal and to identify the determinants of its nonuse and underuse by examining data from the National Health Survey 2014.

**Methods:**

We analyzed data on 8,758 women aged 30 years or older. We defined women at an eligible age for mammography as women aged 45 to 69. Women who reported a previous mammography test were classified as ever-users and grouped according to time since the most recent test. We computed the prevalence of mammography use, and we used Poisson regression models to obtain age-adjusted and education-adjusted prevalence ratios and 95% confidence intervals.

**Results:**

The overall prevalence of mammography use was 80.0%, whereas nonuse was 20.0% and underuse 27.3% among users. The prevalence of nonuse and underuse were lower and associations with sociodemographic characteristics, use of health care services, and behavioral factors were stronger among women aged 45 to 69 than among women aged 30 to 44 and women aged 70 or older. The prevalence of mammography use was generally higher in the northern areas of Portugal than in southern areas and varied by marital status, educational level, and household size. A more frequent use of health care services and healthier behaviors were associated with lower prevalences of both nonuse and underuse.

**Conclusion:**

This study illustrates inequalities in mammography use and provides useful information for better allocation of resources in breast cancer screening.

## Introduction

Breast cancer is the most frequent cancer among women in the world (1). It ranks first as cause of cancer death among women in less developed regions and second as cause of cancer death in more developed regions (2). Nevertheless, 5-year survival has increased worldwide, surpassing 80% in several high-income countries (3). Besides the availability of more effective treatments, the widespread use of mammography screening is also responsible for these improvements (4).

The European Council recommends the screening of women aged 50 to 69 years through mammography every 2 years; organized screening programs (ie, population-based screening programs aimed at the entire resident population of a given age range and sex, designed to detect malignant disease during the detectable preclinical phase [before the occurrence of symptoms], organized at national or regional level, with an explicit policy, a team responsible for organization and for health care, and a structure for quality assurance [5]) are intended to reduce inequalities in access to early detection (6). Although these organized screening programs have been implemented in nearly all European Union countries, screening discrepancies exist, namely in coverage (ie, the proportion of the targeted population invited to have a screening according to the scheduled policy) and rates of participation (ie, the number of women who have a screening test as a proportion of all women who are invited to have a screening) (5,7). In Portugal (8), regional screening programs have been implemented in Centro since 1990, in Lisboa e Vale do Tejo since 1991, and in Alentejo since 1997; programs started later in Algarve (2005) and in Norte (2009). Except for in Algarve, where the Algarve Oncology Association (Associação Oncológica do Algarve) is responsible for the organized screening of women aged from 50 to 69, the other regional programs are conducted by the Portuguese Cancer League (Liga Portuguesa Contra o Cancro) and target women aged 45 to 69 years. Nationally, geographic coverage has been increasing, from about 55% in 2009 to 72% in 2015 (8), whereas adherence to screening has slightly declined recently, from 64.5% in 2012 to 62% in 2015, and regional differences exist.

An evaluation of data from Portugal’s National Health Survey 2005–2006 concluded that the use of mammography among eligible women (ie, women aged 45 to 69) varied by region, sociodemographic characteristics, and access to and use of health care services (9). Understanding the patterns of mammography use and monitoring changes in use is essential to improving national health policy for breast cancer control. This study aimed to describe the use of mammography by women in Portugal and to identify the determinants of its nonuse and underuse by examining data from the National Health Survey (NHS) 2014.

## Methods

Our analysis was based on data collected as part of the NHS 2014, which is a community-based cross-sectional study that evaluated a sample, obtained through multistage stratified and cluster sampling, of the population living in Portugal.

A sample of households was defined by using data from the 2011 Population and Housing Census; the sample was used as the sampling frame for household surveys conducted by Statistics Portugal. The sample consisted of 1,183 primary sampling units, selected systematically from larger geographical strata; the probability of selecting a primary sampling unit was proportional to the number of households in each unit. A random sample of the households was then selected, and all people aged 15 years or older living in these households at the date of the recruitment were eligible to participate. In each household, the selected person was the one whose previous birthday was closest to the date of the contact. The sample size was defined to ensure a homogeneous distribution of the participants among the 7 regions classified by NUTS (Nomenclature of Territorial Units for Statistics) Level 2 (http://ec.europa.eu/eurostat/documents/345175/7451602/nuts-map-PT.pdf). The NUTS classification system is a hierarchical system for dividing the economic territory of the European Union into regions for the purpose of collecting and analyzing data (10).

From September through December 2014, 22,538 households were approached and 18,204 people (56.4% women) were surveyed. All information was collected by using either computer-assisted personal interviewing or computer-assisted web interviewing (50% in each stratum). The questionnaire covered 4 thematic areas: health status, health care, health determinants, and income and health expenses. Use of mammography testing was assessed in a section on preventive care by asking 2 questions: “Have you ever had a mammography (breast x-ray)?” and, if yes, “When was the last time?” The latter question was followed by the response options “in the last 12 months,” “between 1 year and less than 2 years,” “between 2 years and less than 3 years,” and “3 years or more.” A total of 10,240 women provided information on mammography use. We restricted our analyses to women aged 30 years or older (n = 9,119). We further excluded data on 361 women for whom data were missing on the determinants of interest; our final sample consisted of 8,758 women. Women who reported having a previous mammogram were classified as ever-users and grouped further according to the time since the latest test: up to 2 years (including the options “in the last 12 months,” “between 1 year and less than 2 years,”) or 2 or more years (including the options “between 2 years and less than 3 years,” and “3 years or more”). The latter category (2 or more years) was defined as underuse.

We computed the prevalence of mammography testing and used Poisson regression models to compute age-adjusted and education-adjusted prevalence ratios (PRs) and 95% confidence intervals (CIs) to assess the determinants of mammography nonuse and underuse. All analyses were conducted with Stata version 11.2 (StataCorp LP) by using sampling weights computed according to design weight (ie, the inverse of the probability of selection of each primary sampling unit and of each household in each primary sampling unit, further corrected for nonresponse and for the effective number of participants evaluated, and taking into account the age- and sex-structures). For the determinants of nonuse, analyses were further stratified by age group. The analyses on determinants of underuse were restricted to the age group most commonly targeted by organized screening programs in Portugal (women aged 45 to 69).

## Results

Nearly half of the women in the study sample were aged 45 to 69 and therefore potentially eligible for breast cancer screening (Table 1). By education level, 14.5% had not completed a basic level and 18.7% had completed more than a than secondary level. Nearly 20% of the women had access to health care also through other subsystems besides the national health system, and 17.8% had a private health insurance. 

**Table 1:** Characteristics of the Sample and Prevalence of Mammography Use Among Women Aged ≥30, National Health Survey, Portugal, 2014

Characteristics	No.	Unweighted %	Weighted %^a^	Prevalence of Mammography Use, % (95% CI)
**Overall**	8,758	—	—	80.0 (78.8-81.3)
**Region of residence (NUTS 2)**				
Norte	1,326	15.1	34.8	79.9 (77.4–82.3)
Centro	1,800	20.6	22.1	81.7 (79.5–83.8)
Lisboa	971	11.1	27.2	83.8 (81.1–86.4)
Alentejo	1,332	15.2	7.3	75.1 (72.4–77.8)
Algarve	1,231	14.1	4.2	69.6 (66.6–72.6)
RA Açores	947	10.8	2.0	65.8 (62.2–69.3)
RA Madeira	1,151	13.1	2.4	70.9 (67.8–74.0)
**Degree of urbanization^b^**				
Densely populated area	2,669	30.5	44.2	82.9 (80.9–84.9)
Intermediate density area	2,872	32.8	28.8	77.6 (75.3–80.0)
Thinly populated area	3,217	36.7	26.9	77.9 (75.7–80.1)
**Nationality**				
Portuguese	8,577	97.9	98.1	80.3 (79.0–81.5)
Other	181	2.1	1.9	68.2 (58.3–78.0)
**Age, y**				
30–44	2,253	25.7	30.1	54.9 (52.0–57.8)
45–49	774	8.8	10.3	94.4 (92.2–96.5)
50–54	818	9.3	10.4	97.2 (95.8–98.5)
55–59	790	9.0	9.6	96.1 (94.2–98.1)
60–64	869	9.9	8.8	97.9 (96.6–99.2)
65–69	846	9.7	8.2	95.3 (93.2–97.4)
≥70	2,408	27.5	22.5	79.8 (77.3–82.3)
**Legal marital status**				
Single	1,273	14.5	13.7	59.4 (55.1–63.6)
Married	4,541	51.8	63.3	83.5 (82.1–85.0)
Divorced	965	11.0	8.9	86.8 (83.6–90.0)
Widowed	1,979	22.6	14.1	80.2 (77.3–83.0)
**Education level**				
No basic level completed	1,557	17.8	14.5	76.7 (73.5–80.0)
Second basic level completed	3,555	40.6	37.7	89.0 (87.4–90.5)
Third basic level completed	1,056	12.1	13.6	78.5 (74.9–82.0)
Secondary level completed	1,168	13.3	15.5	73.6 (70.0–77.1)
Higher than secondary level completed	1,422	16.2	18.7	71.2 (67.8–74.6)
**Employment status**				
Employed	3,520	40.2	45.2	75.5 (73.5–77.6)
Unemployed	834	9.5	11.1	74.0 (69.8–78.2)
Retired or disabled	3,274	37.4	31.3	85.8 (84.0–87.7)
Housewife	1,130	12.9	12.3	87.3 (84.4–90.2)
**No. of people living in household, including respondent**				
1	2,533	28.9	13.9	78.4 (76.1–80.7)
2	3,072	35.1	32.9	87.0 (85.4–88.6)
3	1,678	19.2	28.0	76.6 (74.0–79.4)
4	1,089	12.4	17.6	76.8 (73.4–80.1)
>4	386	4.4	7.5	72.8 (66.5–79.0)
**Household income per adult**				
Quintile 1	2,025	23.1	20.7	78.3 (75.6–81.1)
Quintile 2	1,871	21.4	20.7	79.6 (77.0–82.2)
Quintile 3	1,807	20.6	20.7	80.9 (78.1–83.6)
Quintile 4	1,515	17.3	18.5	80.2 (77.2–83.1)
Quintile 5	1,540	17.6	19.4	81.4 (78.6–84.2)
**Public health care provider**				
Only national health system	7,039	80.4	80.8	78.1 (76.7–79.5)
National health system and other subsystem	1,719	19.6	19.2	88.2 (86.0–90.4)
**Private health insurance**				
No	7,456	85.1	82.2	80.8 (79.5–82.1)
Yes	1,302	14.9	17.8	76.5 (73.3–79.8)
**Self-perceived health status**				
Very poor	467	5.3	4.9	86.0 (83.5–88.6)
Poor	1,433	16.4	14.3	86.6 (85.0–88.2)
Fair	3,895	44.5	42.9	87.9 (83.6–92.2)
Good	2,409	27.5	31.0	70.9 (68.3–73.6)
Very good	554	6.3	6.9	62.4 (56.4–68.3)
**Diagnosis of chronic disease**				
No	2,525	28.8	32.2	71.4 (68.8–73.9)
Yes	6,233	71.2	67.8	84.2 (82.8–85.5)
**Most recent appointment with general practitioner**				
More than 1 year ago (includes never)	1,725	19.7	17.4	69.6 (66.3–73.0)
In the past year	7,033	80.3	82.6	82.2 (80.9–83.6)
**Last appointment with specialist physician**				
More than 1 year ago (includes never)	4,125	47.1	44.3	77.3 (75.4–79.2)
In the past year	4,633	52.9	55.7	82.2 (80.6–83.8)
**Cervical cytology test**				
Never	2,771	31.6	21.6	66.3 (63.4–62.3)
Ever (at least once)	5,987	68.4	78.4	83.8 (82.5–85.2)
**Fecal occult blood test and/or colonoscopy**				
Never	4,967	56.7	52.2	71.0 (69.1–73.0)
Ever (at least once)	3,791	43.3	47.8	89.9 (88.5–91.3)
**Body mass index^c^**				
<18.5	148	1.7	1.6	65.2 (53.4–76.9)
18.5–24.9	3,574	40.8	43.6	74.9 (72.8–77.0)
25.0–29.9	3,168	36.2	35.3	83.6 (81.7–85.4)
≥30.0	1,868	21.3	19.5	86.4 (84.2–88.7)
**No. of portions of fruits and vegetables per day**				
<5	6,989	79.8	77.7	78.8 (77.3–80.2)
≥5	1,769	20.2	22.3	84.5 (82.1–86.9)
**Smoking status**				
Never	6,695	76.5	74.7	81.1 (79.6–82.5)
Former	1,020	11.6	13.3	80.1 (76.6–83.6)
Current	1,043	11.9	12.0	73.7 (69.8–77.6)
**Drinking status**				
Never	3,406	38.9	31.8	80.0 (78.0–82.0)
Former	999	11.4	12.3	79.3 (75.6–83.1)
Current	4,353	49.7	55.9	80.2 (78.5–81.9)

Abbreviations: CI, confidence interval; NUTS 2, Nomenclature of Territorial Units for Statistics, level 2; RA, Região Autónoma (autonomous region).

a Sampling weights were computed according to design weight (ie, the inverse of the probability of selection of each primary sampling unit and of each household in each primary sampling unit, further corrected for nonresponse and for the effective number of participants evaluated, and taking into account the age and sex structures).

b Based on the share of local population living in urban clusters and in urban centers according to Commission Directorates-General for Regional and Urban Policy, Agriculture and Rural Development, Eurostat, Joint Research Centre, and the Organisation for Economic Co-operation and Development.

c Self-reported weight and height were used to compute body mass index (kg/m^2^), which was divided into 3 categories according to the World Health Organization guidelines (11).

The overall prevalence of mammography use was 80.0% (95% CI, 78.8%–81.3%), whereas nonuse was 20.0% (95% CI, 18.7%–21.2%) (Figure). Of women eligible for breast cancer screening in 2014, 96.2% (95% CI, 95.3%–97.0%) had ever received a mammogram, and 3.8% (95% CI, 3.0%–4.6%) had never had one. By age group, the lowest prevalences of nonuse were among women aged 45 to 69, ranging from 2.1% (95% CI, 0.8%–3.4%) for women aged 60 to 64 years to 5.6% (95% CI, 3.5%–7.7%) for women aged 45 to 49. Among ever-users, the prevalence of underuse was 27.3% (95% CI, 25.7%–28.8%); the prevalence was lowest among women aged 45 to 69 (13.1% [95% CI, 11.5%–14.7%]), ranging from 10.2% (95% CI, 7.5%–13.0%) for women aged 60 to 64 years to 16.2% (95% CI, 12.5%–19.9%) for women aged 65 to 69.

**Figure:**
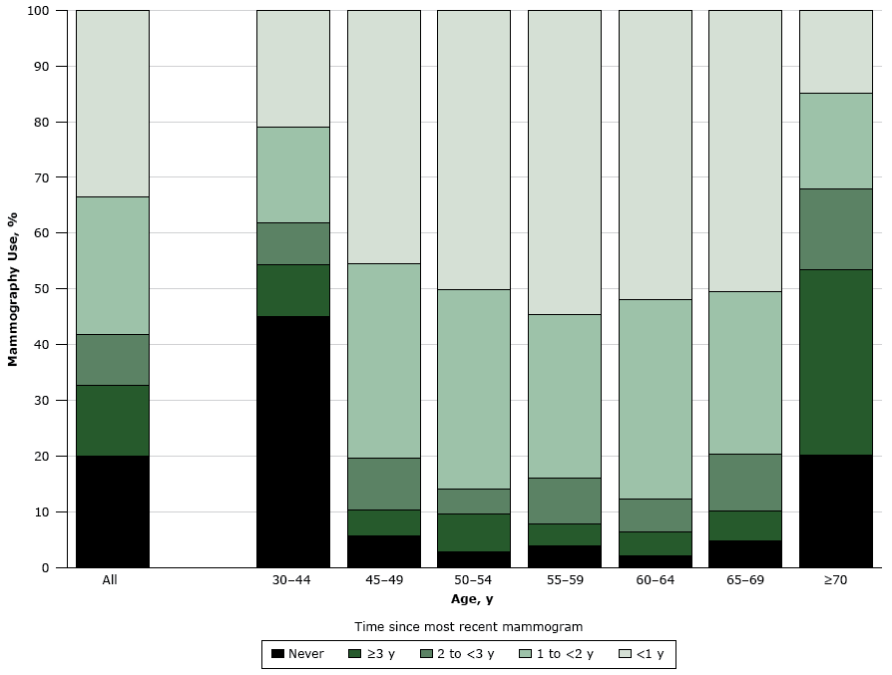
Prevalence of mammography use in Portugal among women aged 30 years or older, according to the elapsed time since most recent test, by age group. Data are from the National Health Survey 2014. **Table:** 

Time Since Most Recent Test	All Ages	30–44 y	45–49 y	50–54 y	55–59 y	60–64 y	65–69 y	≥70 y
Never	20.0	45.1	5.6	2.8	3.9	2.1	4.7	20.2
≥3 y	12.6	9.2	4.7	6.8	3.9	4.2	5.4	33.3
2 y to <3 y	9.2	7.8	9.3	4.6	8.4	5.9	10.1	14.6
1 y to <2 y	24.7	17.0	35.0	35.8	29.3	35.8	29.3	17.1
<1 y	33.5	20.9	45.4	50.1	54.6	52.0	50.6	14.8

Women aged 45 or older living in Alentejo, Algarve, RA Açores, or RA Madeira were more likely than similarly aged women living in Norte to never have had a mammogram (Table 2). Among women aged 30 to 44, compared with women living in Norte, women living in RA Açores were more likely to never have had a mammogram, but women living in Lisboa were less likely to never have had a mammogram. Among women aged 70 or older, the prevalence of mammography nonuse was higher among women living in areas of intermediate density or thinly populated areas than among women in densely populated areas. Among women aged 45 to 69, non-Portuguese women were more likely than Portuguese women to never have had a mammogram.

**Table 2:** Determinants of Mammography Nonuse Among Women Aged 30 Years or Older, by Age Group, Portugal, 2014

Characteristic	Aged 30–44 (n = 2,253)		Aged 45–69 (n = 4,097)		Aged ≥ 70 (n = 2,408)	
	%	Adjusted PR^a^ (95% CI)	%	Adjusted PR^a^ (95% CI)	%	Adjusted PR^a^ (95% CI)
**Region of residence (NUTS 2)**						
Norte	48.7	1 [Reference]	2.9	1 [Reference]	19.0	1 [Reference]
Centro	42.8	0.87 (0.74–1.01)	3.2	1.06 (0.54–2.08)	19.5	0.99 (0.71–1.36)
Lisboa	36.1	0.72 (0.59–0.87)	3.4	1.24 (0.59–2.61)	14.6	0.85 (0.55–1.30)
Alentejo	51.6	1.04 (0.88–1.23)	6.1	2.13 (1.17–3.86)	29.4	1.39 (1.03–1.88)
Algarve	56.8	1.14 (0.97–1.32)	9.6	3.29 (1.89–5.75)	35.6	1.82 (1.36–2.45)
RA Açores	66.2	1.32 (1.14–1.51)	8.2	2.87 (1.56–5.27)	35.4	1.88 (1.31–2.68)
RA Madeira	57.2	1.16 (0.99–1.35)	8.7	2.88 (1.62–5.10)	32.4	1.64 (1.19–2.25)
**Degree of urbanization^b^**						
Densely populated area	41.3	1 [Reference]	3.4	1 [Reference]	14.0	1 [Reference]
Intermediate density area	48.7	1.15 (1.00–1.33)	4.0	1.18 (0.69–2.02)	21.6	1.58 (1.14–2.20)
Thinly populated area	47.3	1.11 (0.96–1.29)	4.4	1.18 (0.70–2.00)	26.9	1.75 (1.30–2.35)
**Nationality**						
Portuguese	45.1	1 [Reference]	0.4	1 [Reference]	20.2	1 [Reference]
Other	45.1	0.97 (0.71–1.32)	14.5	3.84 (1.63–9.05)	—	—
**Legal marital status**						
Married	42.7	1 [Reference]	2.6	1 [Reference]	12.9	1 [Reference]
Single	57.4	1.06 (0.92–1.20)	15.4	6.06 (3.82–9.62)	30.5	1.54 (1.00–2.35)
Divorced	32.1	0.80 (0.62–1.01)	2.7	1.15 (0.64–2.06)	6.4	0.67 (0.30–1.47)
Widowed	24.9	0.90 (0.38–2.12)	3.7	1.39 (0.72–2.70)	26.5	1.36 (1.00–1.83)
**Educational level**						
Higher than secondary level completed	48.7	1 [Reference]	3.0	1 [Reference]	2.1	1 [Reference]
Secondary level completed	44.1	0.95 (0.82–1.11)	3.2	1.11 (0.50–2.46)	22.6	10.27 (3.16–33.37)
Third basic level completed	45.2	0.95 (0.80–1.13)	5.2	1.81 (0.83–3.92)	10.1	4.39 (1.38–13.96)
Second basic level completed	38.0	0.95 (0.78–1.15)	3.3	1.28 (0.66–2.50)	15.5	7.00 (2.66–18.40)
No basic level completed	71.8	1.49 (1.05–2.12)	8.2	3.29 (1.41–7.69)	26.3	10.12 (3.90–26.28)
**Employment status**						
Employed	44.8	1 [Reference]	3.6	1 [Reference]	18.6	1 [Reference]
Unemployed	46.6	1.03 (0.89–1.20)	6.4	1.82 (1.01–3.38)	—	—
Retired or disabled	41.8	0.83 (0.46–1.50)	2.8	0.64 (0.26–1.55)	20.2	1.07 (0.17–6.79)
Housewife	45.5	1.00 (0.70–1.42)	4.3	1.09 (0.56–2.12)	20.2	1.17 (0.18–7.54)
**No. of people living in household, including respondent**						
2	50.9	1 [Reference]	3.1	1 [Reference]	14.6	1 [Reference]
1	54.7	1.07 (0.88–1.30)	5.4	1.77 (1.10–2.84)	25.0	1.34 (1.04–1.71)
3	47.5	0.93 (0.80–1.09)	3.5	1.07 (0.55–2.10)	25.6	1.28 (0.86–1.92)
4	37.1	0.88 (0.74–1.05)	5.0	1.51 (0.71–3.21)	18.9	1.15 (0.50–2.62)
>4	47.9	1.01 (0.77–1.32)	4.9	1.57 (0.66–3.75)	24.2	1.59 (0.77–3.29)
**Household income per adult**						
Quintile 5	42.1	1 [Reference]	2.9	1 [Reference]	11.3	1 [Reference]
Quintile 4	43.8	1.04 (0.86–1.25)	3.8	1.27 (0.53–3.03)	11.6	0.72 (0.33–1.57)
Quintile 3	42.9	1.13 (0.92–1.39)	2.9	0.93 (0.39–2.18)	18.6	1.08 (0.57–2.06)
Quintile 2	53.0	1.42 (1.18–1.72)	3.4	1.07 (0.43–2.67)	21.2	1.24 (0.66–2.35)
Quintile 1	46.4	1.26 (1.01–1.57)	6.0	1.78 (0.72–4.40)	28.8	1.53 (0.81–2.89)
**Public health care provider**						
National health system and other subsystem	33.3	1 [Reference]	2.9	1 [Reference]	10.2	1 [Reference]
Only national health system	47.4	1.29 (1.06–1.58)	4.1	1.18 (0.56–2.47)	21.8	1.27 (0.74–2.17)
**Private health insurance**						
Yes	43.2	1 [Reference]	3.3	1 [Reference]	9.5	1 [Reference]
No	45.9	1.10 (0.96–1.26)	4.0	1.11 (0.56–2.21)	20.6	0.97 (0.50–1.88)
**Self-perceived health status**						
Fair	38.7	1 [Reference]	3.0	1 [Reference]	17.8	1 [Reference]
Very poor	27.7	0.72 (0.33–1.57)	1.0	0.29 (0.11–0.76)	17.2	0.89 (0.61–1.30)
Poor	23.8	0.66 (0.40–1.10)	2.2	0.65 (0.31–1.36)	23.6	1.17 (0.90–1.51)
Good	48.1	1.13 (0.97–1.32)	5.5	2.12 (1.27–3.53)	24.2	1.44 (0.99–2.10)
Very good	51.6	1.19 (0.96–1.47)	10.3	4.47 (2.04–9.80)	21.5	1.46 (0.77–2.77)
**Diagnosis of chronic disease**						
Yes	42.4	1 [Reference]	2.8	1 [Reference]	20.1	1 [Reference]
No	47.3	1.04 (0.92–1.18)	6.5	2.54 (1.56–4.13)	20.7	1.27 (0.88–1.81)
**Most recent appointment with general practitioner**						
In the past year	43.7	1 [Reference]	2.8	1 [Reference]	18.1	1 [Reference]
More than 1 year ago (includes never)	49.4	1.16 (1.01–1.32)	9.2	3.27 (2.12–5.04)	39.5	2.01 (1.58–2.54)
**Most recent appointment with specialist physician**						
In the past year	41.8	1 [Reference]	2.2	1 [Reference]	15.7	1 [Reference]
More than 1 year ago (includes never)	49.8	1.22 (1.08–1.39)	5.7	2.42 (1.53–3.83)	26.0	1.52 (1.21–1.92)
**Cervical cytology test**						
Ever (at least once)	43.2	1 [Reference]	1.4	1 [Reference]	3.6	1 [Reference]
Never	61.8	1.28 (1.08–1.52)	17.0	12.23 (7.86–19.03)	36.8	7.76 (4.42–13.61)
**Fecal occult blood test and/or colonoscopy**						
Ever (at least once)	36.9	1 [Reference]	1.3	1 [Reference]	13.1	1 [Reference]
Never	47.7	1.20 (1.02–1.42)	7.2	5.18 (3.03–8.88)	30.8	2.12 (1.66–2.71)
**Body mass index^c^**						
18.5–24.9	47.3	1 [Reference]	3.8	1 [Reference]	23.2	1 [Reference]
<18.5	39.6	0.87 (0.54–1.41)	16.2	4.20 (1.42–12.39)	45.5	1.67 (0.97–2.87)
25.0–29.9	41.5	0.96 (0.83–1.12)	4.0	1.04 (0.62–1.74)	19.0	0.91 (0.71–1.18)
≥30.0	43.6	1.02 (0.84–1.24)	3.1	0.78 (0.46–1.35)	14.4	0.71 (0.51–1.00)
**No. of portions of fruits and vegetables per day**						
≥5	42.4	1 [Reference]	2.2	1 [Reference]	13.5	1 [Reference]
<5	45.8	1.01 (0.87–1.18)	4.4	1.90 (1.00–3.61)	21.4	1.17 (0.82–1.69)
**Smoking status**						
Never	45.1	1 [Reference]	3.9	1 [Reference]	20.9	1 [Reference]
Former	42.0	0.90 (0.76–1.07)	2.5	0.66 (0.32–1.33)	8.9	0.64 (0.30–1.39)
Current	48.0	1.06 (0.92–1.22)	4.8	1.24 (0.67–2.27)	4.5	0.32 (0.10–1.08)
**Drinking status**						
Never	44.3	1 [Reference]	4.3	1 [Reference]	21.8	1 [Reference]
Former	46.8	1.04 (0.83–1.29)	2.3	0.50 (0.28–0.88)	24.0	1.12 (0.84–1.51)
Current	45.2	1.04 (0.90–1.19)	3.9	0.95 (0.61–1.47)	16.8	0.81 (0.62–1.06)

Abbreviations: CI, confidence interval; NUTS 2, Nomenclature of Territorial Units for Statistics, level 2; PR, prevalence ratio; RA, Região Autónoma (autonomous region).

a Adjusted for age and education, except in education strata.

b Based on the share of local population living in urban clusters and in urban centers according to Commission Directorates-General for Regional and Urban Policy, Agriculture and Rural Development, Eurostat, Joint Research Centre, and the Organisation for Economic Co-operation and Development.

c Self-reported weight and height were used to compute body mass index (kg/m^2^), which was divided into 3 categories according to the World Health Organization guidelines (11).

Single women aged 45 to 69 were more likely than married women of that age to have never had a mammogram. By education level, the prevalence of mammography nonuse was significantly higher only among women with no basic educational level completed, compared with the highest level of education, except among women aged 70 or older, for whom the prevalence of nonuse was higher at all levels of education, compared with the highest level. Unemployed women at the time of the interview were more likely than employed women to have never had a mammogram in the age group 45 to 69. The prevalence of mammography nonuse was higher among women aged 45 or older living alone compared with similarly aged women in a household of 2 people. In the age group 30 to 44, the prevalence of nonuse was significantly higher among women in the 2 lowest quintiles of monthly household income per adult.

Women using only the NHS as their public health care provider were more likely to have never had a mammogram in the group aged 30 to 44, but we found no differences between women with private insurance and women without a private health insurance in any age group. In the age group 45 to 69, women who perceived their health status as very poor were less likely than women who perceived their health as fair to have never had a mammogram, while the opposite was observed for those who considered their health status as good or very good. In this same age group, women without a previous diagnosis of chronic disease were more likely to have never had a mammogram. The prevalence of mammography nonuse was significantly higher among women who had their most recent appointment with a general practitioner or a specialist physician more than one year before the interview and among who had never had a cervical cytology test or fecal occult blood test and/or colonoscopy in all age groups. Among women aged 45 to 69 years, the prevalence of mammography nonuse was higher among underweight women, compared with normal-weight women, and lower in former drinkers, compared with never-drinkers.

Women aged 45 to 69 living in Lisboa, Alentejo, Algarve, RA Açores, and RA Madeira were significantly more likely than similarly aged women living in Norte to underuse mammography; so were non-Portuguese women, compared with Portuguese women (Table 3). Single and divorced women, compared with married women, as well as those living in households consisting of more than 4 people were more likely to underuse mammography. By education level, the prevalence of mammography underuse was the lowest among women who completed a second basic educational level.

**Table 3:** Determinants of Mammography Underuse^a^ Among Women Aged 45–69 Years (n = 4,097) Who Reported Having Had a Previous Mammography, Portugal, 2014

Characteristic	%	Adjusted PR^b^ (95% CI)
**Region of residence (NUTS 2)**		
Norte	8.4	1 [Reference]
Centro	12.5	1.45 (1.00–2.10)
Lisboa	17.4	1.95 (1.35–2.83)
Alentejo	14.8	1.74 (1.22–2.48)
Algarve	20.5	2.40 (1.70–3.39)
RA Açores	14.6	1.73 (1.19–2.52)
RA Madeira	22.8	2.65 (1.89–3.71)
**Degree of urbanization^c^**		
Densely populated area	13.4	1 [Reference]
Intermediate density area	12.0	0.97 (0.72–1.30)
Thinly populated area	13.8	1.12 (0.84–1.48)
**Nationality**		
Portuguese	12.8	1 [Reference]
Other	30.8	2.34 (1.30–4.23)
**Legal marital status**		
Married	11.2	1 [Reference]
Single	22.1	1.87 (1.31–2.68)
Divorced	17.3	1.52 (1.05–2.20)
Widowed	15.4	1.36 (0.96–1.92)
**Educational level**		
Higher than secondary level completed	16.4	1 [Reference]
Secondary level completed	14.3	0.89 (0.56–1.42)
Third basic level completed	13.0	0.80 (0.52–1.22)
Second basic level completed	11.0	0.68 (0.48–0.97)
No basic level completed	19.2	1.13 (0.70–1.84)
**Employment status**		
Employed	13.4	1 [Reference]
Unemployed	15.7	1.26 (0.86–1.87)
Retired or disabled	12.8	0.78 (0.52–1.18)
Housewife	10.9	0.81 (0.55–1.20)
**No. of people living in household, including respondent**		
2	12.7	1 [Reference]
1	14.7	1.08 (0.82–1.43)
3	13.3	1.04 (0.76–1.43)
4	8.7	0.64 (0.39–1.06)
>4	22.2	1.79 (1.08–2.96)
**Household income per adult**		
Quintile 5	12.2	1 [Reference]
Quintile 4	13.7	1.39 (0.89–2.18)
Quintile 3	12.5	1.37 (0.83–2.25)
Quintile 2	13.1	1.47 (0.89–2.42)
Quintile 1	13.9	1.61 (0.97–2.68)
**Public health care provider**		
National health system and other subsystem	13.3	1 [Reference]
Only national health system	13.0	1.17 (0.82–1.68)
**Private health insurance**		
Yes	12.8	1 [Reference]
No	13.1	1.14 (0.80–1.61)
**Self-perceived health status**		
Fair	12.0	1 [Reference]
Very poor	14.0	1.12 (0.64–1.97)
Poor	11.3	0.94 (0.65–1.35)
Good	13.9	1.11 (0.80–1.55)
Very good	25.7	2.01 (1.28–3.15)
**Diagnosis of chronic disease**		
Yes	12.4	1 [Reference]
No	14.8	1.14 (0.86–1.52)
**Most recent appointment with general practitioner**		
In the past year	10.5	1 [Reference]
More than 1 year ago (includes never)	27.1	2.50 (1.91–3.27)
**Most recent appointment with specialist physician**		
In the past year	10.4	1 [Reference]
More than 1 year ago (includes never)	16.3	1.62 (1.26–2.08)
**Cervical cytology test**		
Ever (at least once)	11.6	1 [Reference]
Never	22.8	1.98 (1.52–2.57)
**Fecal occult blood test and/or colonoscopy**		
Ever (at least once)	9.8	1 [Reference]
Never	17.8	1.82 (1.43–2.33)
**Body mass index^d^**		
18.5–24.9	13.6	1 [Reference]
<18.5	18.9	1.35 (0.58–3.14)
25.0–29.9	12.8	0.97 (0.72–1.31)
≥30.0	12.6	0.98 (0.70–1.37)
**No. of portions of fruits and vegetables per day**		
≥5	10.3	1 [Reference]
<5	14.1	1.45 (1.06–1.97)
**Smoking status**		
Never	12.0	1 [Reference]
Former	13.4	1.06 (0.72–1.57)
Current	19.4	1.59 (1.12–2.25)
**Drinking status**		
Never	14.6	1 [Reference]
Former	8.3	0.58 (0.36–0.94)
Current	13.2	0.90 (0.69–1.17)

Abbreviations: CI, confidence interval; NUTS 2, Nomenclature of Territorial Units for Statistics, level 2; PR, prevalence ratio; RA, Região Autónoma (autonomous region).

a Most recent mammography performed 2 or more years ago.

b Adjusted for age and education, except in education strata.

c Based on the share of local population living in urban clusters and in urban centers according to Commission Directorates-General for Regional and Urban Policy, Agriculture and Rural Development, Eurostat, Joint Research Centre, and the Organisation for Economic Co-operation and Development.

d Self-reported weight and height were used to compute body mass index (kg/m^2^), which was divided into 3 categories according to the World Health Organization guidelines (11).

Women who perceived themselves as having a very good health were more likely than women who perceived their health as fair to underuse mammography, as were women who had their most recent appointment with a general practitioner or specialist physician more than one year before the interview and those who had never had a cervical cytology test or fecal occult blood test and/or colonoscopy.

The prevalence of mammography underuse was higher among women eating fewer than 5 portions of fruits and vegetables per day, compared with women who ate at least 5 portions, and among current smokers, compared with never-smokers, whereas the opposite was found for former drinkers. Former drinkers were less likely than never-drinkers to underuse mammography.

## Discussion

Our data showed that 96.2% of women eligible for breast cancer screening in 2014 had ever received a mammogram, an increase of approximately 10% (from 87.6%) in mammography use since the 2005–2006 National Health Survey (9). However, a considerable percentage of women have never had a mammogram or did not follow screening. The prevalence of nonuse and underuse was lower among women aged 45 to 69, the age targeted for screening, than among women aged 30 to 44 and women aged 70 or older. Regional variations persisted, and patterns of mammography use differed according to sociodemographic characteristics and access to and use of health care services.

Although the results of our study cannot be generalized to other settings, because our data depended on how breast cancer screening is performed in Portugal, namely through implementation of organized screening programs and local specificities of the health care system, our findings are similar to those reported for other countries using comparable methodologies. Countries such as France (12), Italy (13), and Spain (14) reported similar prevalences of mammography use based on data from recent national health surveys.

We found that rates of mammography nonuse and underuse among women aged 45 to 69 years were higher in the southern regions of Portugal than in the northern region. This finding is in accordance with a recent evaluation of the breast cancer screening program in the northern region of Portugal (15), which showed a participation rate of 74.5% in 2008–2009 and that most performance indicators were consistent with levels defined by the European Guidelines (5). However, a report in 2016 on population-based cancer screening programs in Portugal showed that geographic and population coverage and adherence to screening were at lower levels in the northern region than in other regions of Portugal (approximately 80% and 53% vs 98% and 65%, respectively) (8). Taking into account the report in 2016 and that organized screening in the northern region started later than screening in other regions, the lower prevalence of mammography nonuse in the northern region (2.9% among women aged 45 to 69 years), compared with other regions, found in our study may have been due to a higher prevalence of opportunistic screening in the northern region than in other regions. Opportunistic screening is screening outside an organized or population-based screening program, for example, following a recommendation made during a routine medical consultation, or a consultation for an unrelated condition revealing a potentially increased risk of cancer, or by self-referral. In addition, the territorial units defined by NUTS 2 do not entirely correspond to regions covered by the regional health administration responsible for implementing breast cancer screening programs. Some territorial differences between geographic regions and health regions may translate into an overestimation of mammography use in Lisboa and an underestimation in Centro. Nevertheless, our findings show that regional gaps exist and demonstrate insufficient coverage and/or insufficient participation by eligible women.

Our results are in line with previous evaluations of breast cancer screening uptake by national health surveys, identifying poorer socioeconomic status and lower levels of use of health care services as the main determinants of mammography testing (12,16–19). On one hand, the prevalence of nonuse by education level was highest among women with no basic level of education completed, and we observed no significant differences according to income. Other studies also showed no association between women’s economic situation and adherence to breast cancer screening recommendations (20). On the other hand, greater use of health care services, such as a recent interaction with a usual health care provider and ever having had a cervical cytology test, was associated with low prevalences of mammography nonuse and underuse. Although these associations were also reported in other studies (12,16–19), we additionally found that women who had ever had a fecal occult blood test and/or colonoscopy were also more likely to have received a mammogram. These findings suggest that the closer women are to the health care system, the easier is the process of becoming aware of the importance of monitoring breast cancer and that women should be encouraged to maintain regular contact with their family physicians. Our results also confirmed that potentially easier access to the health care system is associated with a lower risk of not being screened, at least among younger women.

Other sociodemographic characteristics were identified as determinants of mammography use in our study, namely the degree of urbanization among older women. Other studies that account for population density support our findings (12,21). In our study, only marital information on legal marital status was available. Other studies have provided details on women’s living arrangements (eg, living alone, living with partner), regardless of their legal marital status as single women (22). Therefore, a direct comparison of our results with the results of most other studies cannot be made, but our findings on legal marital status are consistent with our findings on household size. Mammography testing was less frequent among non-Portuguese women than among Portuguese women, consistent with the findings of several studies that identified immigrants as a vulnerable population in this context (12,18,19,23).

In our study, the prevalence of mammography nonuse increased as self-perceived health status improved, consistent with a study in Italy (24). These results point to the possible need for additional primary prevention efforts among women who perceive themselves as being healthy in the age group considered to be at higher risk for developing breast cancer (ie, women aged 45 to 69). It was also in this age group that the prevalence of nonuse was higher among women without a diagnosis of chronic disease than among women with such a diagnosis. Other studies reported similar findings (14,16), and, although differences according to the type of chronic disease were described (17,19,25,26), our results may indicate over-screening among women with a chronic disease. Research also consistently shows that obese women are less likely to undergo breast cancer screening than nonobese women (16,18,24–26), but in our study the highest prevalence of nonuse was among underweight women.

Other studies have also reported higher levels of adherence to breast cancer screening among those who have healthy lifestyles, namely women who are physically active (14,17); however, research on the relationship between adherence and diet is less consistent (17). Our study found a significant positive association between mammography underuse and not following recommendations on fruit and vegetables intake (27) but only among women in the age range targeted for screening (aged 45 to 69). In our study, former drinkers were less likely than never-drinkers to underuse or not use mammography; this finding may conflict with those of a study in Spain (14), however, in the latter, only information on current alcohol consumption was provided. Similarly, current smokers were more likely to underuse mammography in our analysis. Only one study showed an association between smoking status and breast cancer screening uptake in line with our results (12); most studies reported no difference in adherence according to this lifestyle factor (24,25).

The main strength of this study is the evaluation of a large representative sample of women living in Portugal. The study provides information on patterns of mammography use and identifies the major determinants of use. These data can be used to improve the national health policy for breast cancer screening. However, this study also has several limitations. Although the 2 questions on mammography use in the NHS 2014 were part of a section referring to preventive care, we cannot ascertain that all women who reported having mammogram had one for screening purposes and not for diagnostic purposes. As a result, we may have underestimated the prevalence of mammography nonuse for screening purposes. Furthermore, even if all women who reported having a mammogram had one for screening purposes, no survey question asked whether the mammogram was received through an organized screening program or through opportunistic screening. This precludes a direct comparison of our results with the results of previous evaluations of organized screening programs and comparisons between organized screening programs and opportunistic screening (13,26). Additionally, no information on personal or family history of breast cancer was collected; this information could have influenced cancer screening initiation and frequency, especially among younger women.

Although our results showed an increase in mammography use in the last 10 years in Portugal, regional differences persist, especially among women in the age range most commonly targeted by organized screening programs conducted at the national level. Differences according to sociodemographic characteristics and access to and use of health care services illustrate inequalities in mammography testing. This study provides useful information for a better allocation of resources for breast cancer screening, namely taking into account the regional and socioeconomic factors identified as possible barriers.
